# Fucosyltransferase 8 is Overexpressed and Influences Clinical Outcomes in Lung Adenocarcinoma Patients

**DOI:** 10.3389/pore.2022.1610116

**Published:** 2022-02-14

**Authors:** Yiqian Liang, Ting Wang, Rui Gao, Xi Jia, Ting Ji, Puyu Shi, Jianjun Xue, Aimin Yang, Mingwei Chen, Peng Han

**Affiliations:** ^1^ Department of Nuclear Medicine, The First Affiliated Hospital of Xi’an Jiaotong University, Xi’an, China; ^2^ Department of Respiratory Medicine, Xi’an No. 4 Hospital, Xi’an, China; ^3^ Department of Respiratory and Critical Care Medicine, The First Affiliated Hospital of Xi’an Jiaotong University, Xi’an, China; ^4^ Department of Otolaryngology-Head and Neck Surgery, The First Affiliated Hospital of Xi’an Jiaotong University, Xi’an, China

**Keywords:** FUT8, lung adenocarcinoma, bioinformatics analysis, prognosis, proliferation

## Abstract

**Background:** Lung adenocarcinoma (LUAD), the most prevalent type of lung cancer, is often metastatic and has a poor prognosis. Recent studies have demonstrated an important role for fucosyltransferase 8 (FUT8) in carcinogenesis and cancer progression.

**Methods:** A meta-analysis with 15 eligible datasets from Gene Expression Omnibus (GEO) was performed to explore the expression of FUT8 in LUAD. The results were further verified in The Cancer Genome Atlas (TCGA) database, followed by survival analysis using Kaplan-Meier plotter. We also validated the protein expression of FUT8 by immunohistochemistry (IHC). *In vitro* experiments were conducted to determine the biological effects of FUT8 in LUAD cells.

**Results:** The meta-analysis showed the FUT8 expression in LUAD tissues was significantly higher than those in normal lung tissues [standard mean difference (SMD): 1.40; 95% confidence interval (CI): .95–1.85]. The results of TCGA database verified the expression of FUT8 increased in LUAD tissues versus normal tissues. IHC analyses indicated that the protein levels of FUT8 were up-regulated in LUAD, and elevated FUT8 expression was significantly correlated with poor prognosis in LUAD patients. Multivariable Cox regression analysis revealed that FUT8 expression was an independent prognostic factor. Besides, *in vitro* experiments showed that knockdown of FUT8 in LUAD cells markedly restrained cell proliferation, and stimulated cell apoptosis.

**Conclusion:** This study indicates that increased FUT8 expression is correlated with shortened survival of LUAD patients and might favor the progression of the disease.

## Introduction

In China, lung cancer is the most common cause of cancer-related deaths in both men and women (1). According to the Global Burden of Disease Study 2017, the mortality for lung cancer increased by 28.2% from 1990 to 2017 in China (2). It is estimated that 774,323 new lung cancer cases occurred in China in 2018, ranking first among all cancer types. Lung adenocarcinoma (LUAD) is the most frequently diagnosed histologic subtype of lung cancer, representing 38.5% of all pulmonary neoplasm cases. Most patients with non-small cell lung cancer (NSCLC) are diagnosed at advanced stages and have a dismal 5-year survival rate of 6.9% (3). Recently, the identification of common driver mutations, such as epidermal growth factor receptor (EGFR) and anaplastic lymphoma kinase, leads to the development of many novel targeted therapies that has profoundly changed the survival outcomes of NSCLC patients, especially those with LUAD (4). However, cancer cells often develop inevitably acquired resistance to these molecular targeted drugs after 10–14 months (5). The emergence of drug resistance leads to unsatisfactory clinical effects of targeted drugs and limit their clinical application. Therefore, it is urgently necessary to identify novel and better therapeutic targets for patients with LUAD.

Fucosylation is the process of transferring a fucose residue from donor guanosine diphosphate (GDP)-fucose to oligosaccharide, glycoprotein, and glycolipid acceptors. Fucosyltransferases (FUTs) mediate the addition of fucose in α1,2 (FUT1 and 2), α1,3 (FUT3-7 and FUT9-11), α1,4 (FUT3 and 5) or α1,6 linkage (FUT8) to N- and O-linked glycans, or lipid-linked glycans (6). Fucose can also be added directly to the Ser and Thr residues of glycoprotein acceptors that contain either the epidermal growth factor (EGF) or the thrombospondin type repeat sequences (protein-O-fucosyltransferase 1 and 2, respectively) (7). Fucosylated glycans have crucial roles in blood transfusion reactions, host-microbe interactions, selectin-dependent leukocyte adhesion, and numerous ontogenic events, including ligand-induced Notch signaling events (8).

Core fucosylation (α1,6-fucosylation) consists of the addition of α1,6-fucose to the innermost N-acetylglucosamine residue of N-glycans carried by the glycoproteins (9). Altered core fucosylation has been implicated in malignant transformation, invasion, and metastasis of certain types of cancer, such as hepatocellular, colorectal, breast and ovarian cancers (10-13). Unlike other FUTs with redundant functions, FUT8 is an enzyme that is uniquely responsible for catalyzing the reaction of core fucosylation. Recently, increasing evidence has supported the crucial implication of α1,6-fucosylation by FUT8 in tumor growth and malignancy, antibody-dependent cellular cytotoxicity, as well as cell adhesion and cancer metastasis (11, 14-17). In our previous study, we found that the serum level of core fucosylation was significantly higher in LUAD patients than in healthy controls, and the levels of core fucosylation in the sera had an increasing trend with clinical stages of LUAD (18). However, the understanding of the prognostic significance and underlying role of FUT8 in LUAD is still limited.

In this study, a meta-analysis was first performed to evaluate the expression pattern and latent diagnostic value of FUT8 in LUAD based on data obtained from Gene Expression Omnibus (GEO). Then, we investigated the contributing role of FUT8 in LUAD by mining The Cancer Genome Atlas (TCGA) datasets and using the program Kaplan-Meier plotter, and FUT8 immunohistochemistry (IHC) staining on LUAD samples was also conducted. Moreover, we performed *in vitro* experiments to investigate the biological function of FUT8 in LUAD.

## Materials and Methods

### Bioinformatics Analysis

To investigate the expression of FUT8 in LUAD, we performed a meta-analysis based on data from GEO (http://www.ncbi.nlm.nih.gov/geo/). The following search terms were used to retrieve eligible datasets from GEO: (lung OR pulmonary) AND (cancer OR carcinoma OR adenocarcinoma OR tumor OR malignanc^*^ OR neoplas^*^). The inclusion criteria were as follows: 1) human species; 2) FUT8 expression was examined in both LUAD tissues and non-cancerous tissues; 3) at least three samples were included in each group. The pooled standard mean difference (SMD) with a 95% confidence interval (CI) was calculated to assess the overall expression level of FUT8 in LUAD. Chi-square test and *I* ^*2*^ statistic were used to evaluate the heterogeneity among included studies. A random-effects model was selected if there was heterogeneity (*I* ^*2*^ > 50% or *p* < .05). Otherwise, a fixed-effects model would be selected (19). The stability of the pooled SMD was assessed by sensitivity analysis. Publication bias was ascertained using Begg’s funnel plot and Egger’s test. A summary receiver operating characteristic (sROC) curve was generated to illustrate the diagnostic ability of FUT8 expression for LUAD.

LUAD RNA-Seq datasets of tumor and nontumor tissues was downloaded from TCGA Data Portal (https://portal.gdc.cancer.gov/). The differences in FUT8 expression between tumor and nontumor groups were analyzed using R language. The prognostic value of FUT8 was analyzed by the meta-analysis tool Kaplan-Meier plotter (20). Univariate Cox regression analysis, Kaplan-Meier survival plot with hazard ratio (HR) and log-rank *p*-value were calculated and plotted.

### Subject Recruitment and Sample Collection

A total of 92 patients with primary LUAD who underwent surgical resection at the First Affiliated Hospital of Xi’an Jiaotong University between January 2008 and July 2013 were enrolled in this study for IHC analysis. They had received no prior radiotherapy and/or chemotherapy, and had available paraffin-embedded specimens. The corresponding adjacent normal tissues were collected from 88 of the 92 LUAD patients. We examined the clinicopathologic features as follows: age at surgical resection, sex, stage, lymph node metastases, differentiation, EGFR mutation status, and overall survival. The study was approved by the ethic committee of the First Affiliated Hospital of Xi’an Jiaotong University (No. XJTU1AF2020LSK-199) and informed consent was obtained from every participant before the initiation of any study-related procedure.

### Immunohistochemistry Analysis

The method used for immunostaining was the streptavidin-biotin amplified system. Briefly, after deparaffinization and rehydration, antigen retrieval was done by heating in Tris-EDTA buffer (pH 9.0) at 97°C followed by cooling at room temperature. Slides were then incubated with 1000-fold diluted anti-human FUT8 monoclonal antibody (Proteintech, Wuhan, China) at 4°C overnight. Subsequently, the biotinylated secondary antibody (Vector Laboratories, Burlingame, CA, United States) was applied to all the sections for 30 min at room temperature, and the sections were washed thrice with PBS, incubated with avidin-biotin-peroxidase complex (Vector Laboratories, United States) for 30 min. Following 3-3′-diaminobenzidine color development, slides were counterstained with hematoxylin.

FUT8 expression was evaluated using the intensity of staining (0 = negative, 1 = weak, 2 = intermediate, and 3 = strong) and the proportion of stained tumor cells per slide (0–100%) as previously reported (21). FUT8 immunostaining was considered to indicate high expression when ≥30% of tumor cells with intermediate or strong staining. Cases with negative, weak, and focal staining with 2 or 3 intensity were considered to indicate low expression. The levels of FUT8 were independently evaluated by two pathologists.

### Cell Culture and Transfection

Human lung adenocarcinoma cell line A549 was obtained from the Chinese Academy of Sciences Cell Bank (Shanghai, China), and cultured in RPMI-1640 medium (Invitrogen, Carlsbad, CA, United States) supplemented with 10% fetal bovine serum (FBS; Sigma-Aldrich, Darmstadt, Germany) and 1% penicillin/streptomycin (Invitrogen). A549 Cells were transfected with FUT8 siRNA or negative control (NC) siRNA using Lipofectamine 2000 reagent (Invitrogen) according to the manufacturer’s instruction. The sequences were si-FUT8 (sense: 5′-GUG​GAG​UGA​UCC​UGG​AUA​UTT-3′, Anti-sense: 5′-AUA​UCC​AGG​AUC​ACU​CCA​CTT-3′), and si-NC (sense: 5′-UUCUCCGAACGUG UCACGUdTdT-3′, Anti-sense: 5′-ACGUGACACGUUCGGAGAAdTdT-3′). After 48 h, the transfection efficiency was verified *via* RT-PCR and western blotting.

### Quantitative Real Time RT-PCR

Total RNA were isolated from harvested cells using TRIzol reagent (Invitrogen). The cDNA was synthesized using PrimeScript RT master mix (Perfect Real Time) (TaKaRa, Shiga, Japan). RT-PCR analysis of FUT8 and β-actin was performed in a StepOnePlus real-time PCR system (Applied Biosystems Inc., Foster City, CA, United States) using SYBR Premix Ex Taq II (Perfect Real Time) (TaKaRa). PCR amplifications were conducted using the following protocol: 95°C for 30 s, for 40 cycles at 95°C for 5s (denaturation step), at 60°C for 30 s (annealing/extension steps). The 2^−ΔΔCT^ method was used to determine differences in FUT8 expression levels between specimens. The primers used in this work were obtained from Sangon Biotech (Shanghai, China) and their sequences were included in Supplementary Table S1.

### Western Blot Analysis

After the indicated treatment, cells were lysed in RIPA buffer (Beyotime, China) containing proteinase inhibitor cocktail (Sigma-Aldrich). The Pierce Coomassie Plus colorimetric protein assay (Thermo Fisher Scientific, Waltham, MA, United States) was used to measure the protein concentration, and approximately 30 μg of total protein was separated by 8% SDS-PAGE and transferred onto nitrocellulose. The membrane was blocked in 5% skim milk for 1 h at room temperature and then overnight at 4°C with primary antibodies against FUT8 (Abcam, Waltham, MA, United States) and β-actin (Sigma-Aldrich). After incubated with HRP-conjugated secondary antibody (Bio-Rad, Hercules, CA, United States), the blots were visualized using the Enhanced Chemiluminescence Detection Kit (GE Healthcare).

### Cell Proliferation and Apoptosis

Cell proliferation was detected by the cell count kit-8 (CCK-8, Dojindo, Japan), according to the manufacturer’s instruction. Briefly, 1 × 10^4^ transfected cells per well were seeded in 96-well plates. After 24, 48, or 72 h of cell growth, 10 μl CCK-8 was added to each well and incubated for 2 h at 37°C. The absorbance value was measured in a microplate reader at 450 nm.

For apoptosis measurement, 1 × 10^5^ transfected cells were seeded in 6-well plates with RPMI-1640 medium containing 10% FBS for 48 h. The cells were then harvested, washed twice with ice-cold phosphate-buffered saline, and re-suspended with binding buffer at a concentration of 1 × 10^6^ cells/ml. Apoptosis was determined by flow cytometry after staining with 7-AAD and annexin V-APC (Keygen Biotech, Nanjing, China).

### Statistical Analysis

The relationship between FUT8 expression and clinicopathological characteristics was analyzed using *χ* ^2^ test for categorical variables and using unpaired *t* test for continuous variables. Overall survival (OS) was defined as the time interval from date of surgery to the date of death, and was censored at last follow-up. Survival data were estimated by the Kaplan-Meier method and compared using the log-rank test. Univariate and multivariate Cox regression analyses for OS were used to identify the significant prognostic factors in LUAD. *p*-values of <.05 were considered statistically significant on the basis of 2-sided testing. All statistical analyses were performed using SPSS 22.0 and GraphPad Prism v5.0.

## Results

### Meta-Analysis of the Association Between Fucosyltransferase 8 Expression and Lung Adenocarcinoma Based on GEO Datasets

**FIGURE 1:**
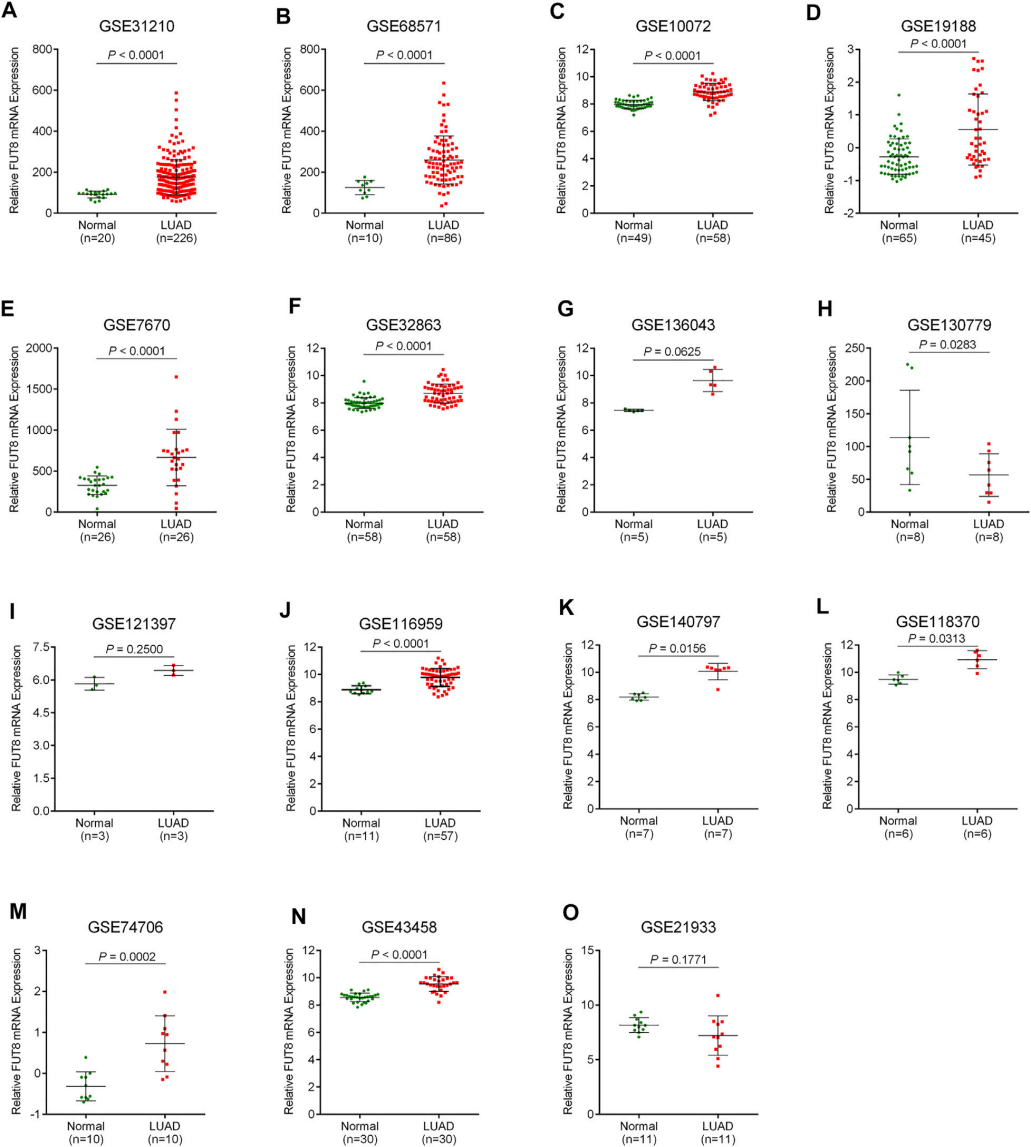
Scatter plots for FUT8 expression in LUAD from GEO datasets. **(A)** GSE31210. **(B)** GSE68571. **(C)** GSE10072. **(D)** GSE19188. **(E)** GSE7670. **(F)** GSE32863. **(G)** GSE136043. **(H)** GSE130779. **(I)** GSE121397. **(J)** GSE116959. **(K)** GSE140797. **(L)** GSE118370. **(M)** GSE74706. **(N)** GSE43458. **(O)** GSE21933. Abbreviations: FUT8, fucosyltransferase 8; LUAD, lung adenocarcinoma.

**FIGURE 2:**
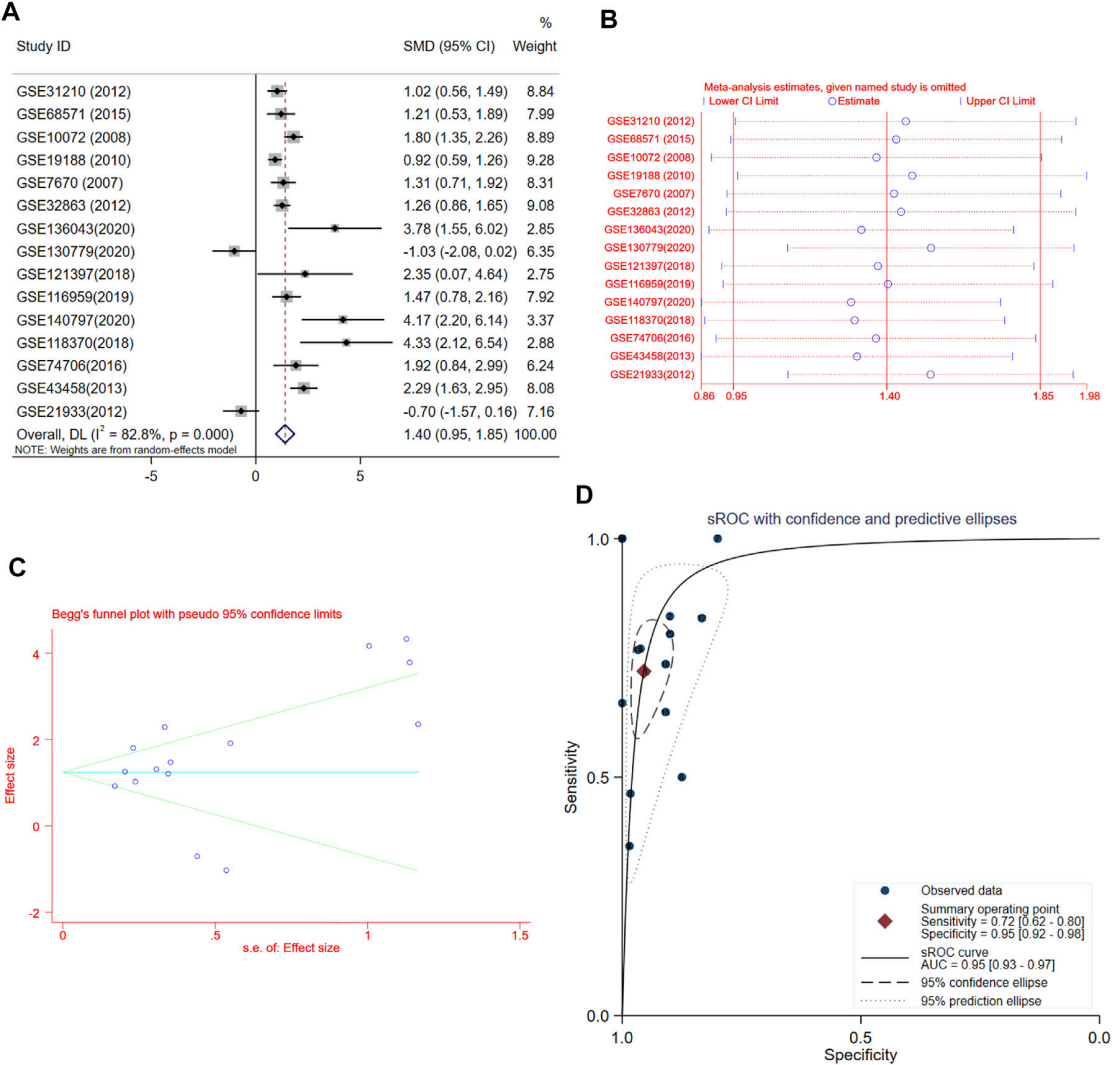
Meta-analysis of FUT8 expression levels in LUAD based on data from GEO. **(A)** Forest plot of 15 records evaluating FUT8 expression in LUAD (random-effects model). If the SMD > 0 and the corresponding 95% CI of the SMD did not overlap zero, FUT8 was upregulated in LUAD tissues compared with normal lung tissues. **(B)** Sensitivity analysis evaluating meta-analysis stability. **(C)** Funnel plot of 15 included studies for FUT8 evaluating potential publication bias (Begg’s method). **(D)** sROC curves for the identification of LUAD patients from normal controls using FUT8 expression. Abbreviations: SMD, standard mean difference; CI, confidence interval; sROC, summary receiver operating characteristic; AUC, area under the curve.

A total of 15 records with FUT8 expression detected in both LUAD samples and control samples were collected in this study. The basic information on these datasets is included in Table 1. The expression of FUT8 in each dataset is presented as scatter plots in Figure 1. Due to the divergences in individual studies, we then combined all of the included datasets. The pooled SMD of FUT8 was 1.40 (95% CI = .95–1.85) by the random-effects model (Figure 2A). The I-squared value was 82.8%, and the *p*-value was less than .001. Sensitivity analysis suggested that the pooled results were stable (Figure 2B). No publication bias was detected in these studies as indicated by Begg’s funnel plot (*p* = .235) and Egger’s test (*p* = .289) (Figure 2C). Simultaneously, sROC curve was also generated to illustrate the ability of FUT8 expression to differentiate LUAD patients from normal controls. As shown in Figure 2D, the area under the curve (AUC) for FUT8 was .95 (95% CI = .93–.97), with a sensitivity and specificity of .72 (95% CI = .62–.80) and .95 (95% CI = .92–.98), respectively.

**TABLE 1:** Main information on the 15 included GEO datasets for FUT8.

Datasets	Region	Year	Platform	Sample size (T/N)	FUT8 expression (mean ± SD)	
					T	N
GSE31210	Japan	2012	GPL570	226/20	175.34 ± 85.16	91.54 ± 16.82
GSE68571	United States	2015	GPL80	86/10	259.10 ± 116.99	124.92 ± 34.67
GSE10072	United States	2008	GPL96	58/49	8.88 ± .63	7.96 ± .30
GSE19188	Netherlands	2010	GPL570	45/65	.55 ± 1.08	-.27 ± .53
GSE7670	Taiwan	2007	GPL96	26/26	665.98 ± 344.82	328.63 ± 113.07
GSE32863	United States	2012	GPL6884	58/58	8.68 ± .69	7.98 ± .39
GSE136043	China	2020	GPL13497	5/5	9.63 ± .81	7.45 ± .08
GSE130779	China	2020	GPL20115	8/8	56.60 ± 32.64	113.87 ± 71.73
GSE121397	China	2018	GPL17586	3/3	6.43 ± .23	5.82 ± .29
GSE116959	France	2019	GPL17077	57/11	9.78 ± .66	8.88 ± .29
GSE140797	China	2020	GPL13497	7/7	10.05 ± .59	8.19 ± .24
GSE118370	China	2018	GPL570	6/6	10.93 ± .33	9.47 ± .34
GSE74706	Germany	2016	GPL13497	10/10	.73 ± .68	-.32 ± .36
GSE43458	United States	2013	GPL6244	30/30	9.54 ± .53	8.56 ± .30
GSE21933	Taiwan	2012	GPL6254	11/11	7.20 ± 1.80	8.17 ± .68

FUT8, fucosyltransferase 8; T, tumor; N, normal; SD, standard deviation.

### Fucosyltransferase 8 Expression Validation in Lung Adenocarcinoma From TCGA Database

**FIGURE 3:**
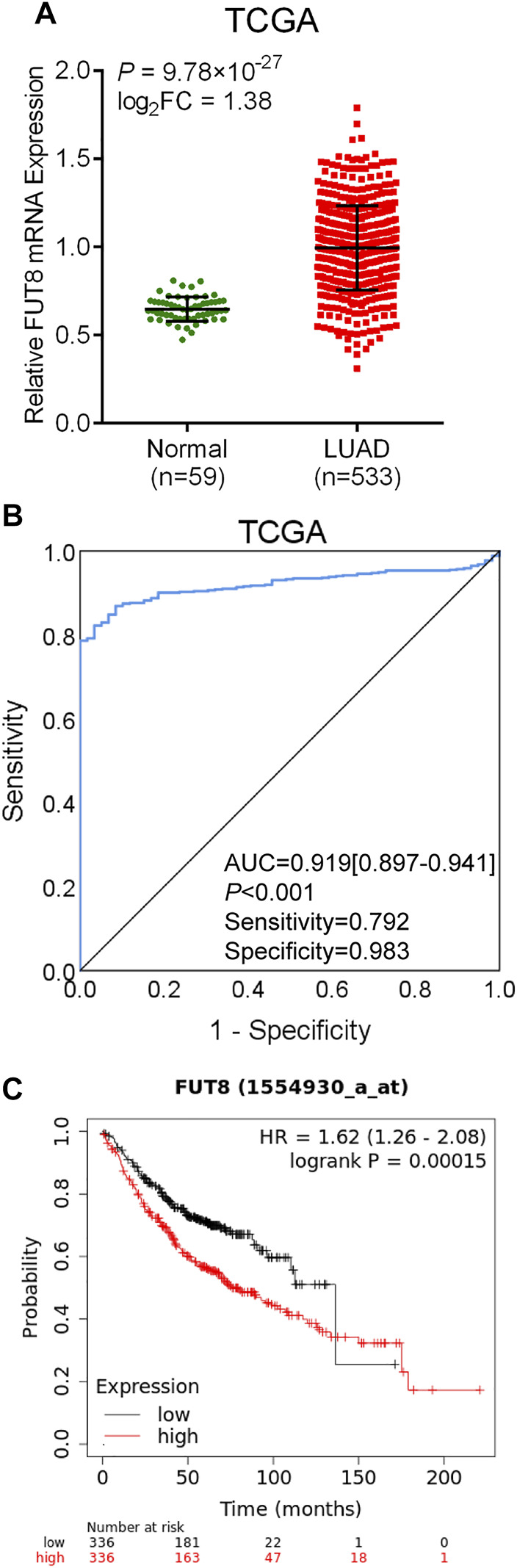
TCGA database analysis of differential expression of the FUT8 gene and prognosis analysis in public databases. **(A)** mRNA expression levels of FUT8 in LUAD tissues and normal tissues obtained from TCGA datasets. **(B)** ROC curve for FUT8 expression in TCGA LUAD. **(C)** Kaplan-Meier plots of overall survival and FUT8 expression in patients with LUAD using the program Kaplan-Meier plotter. Abbreviations: FUT8, fucosyltransferase 8; TCGA, the Cancer Genome Atlas; LUAD, lung adenocarcinoma; AUC, area under the curve; HR, hazard ratio.

The data on 533 LUAD cases (533 cancer tissues and 59 normal lung tissues) with FUT8 expression was extracted from TCGA database. As shown in Figure 3A, the expression level of FUT8 in LUAD tissues was significantly elevated compared with normal tissues in TCGA (log_2_FC = 1.38, *p* = 9.78 × 10^−27^), which was consistent with the result of meta-analysis by GEO datasets. ROC curve was generated to assess the ability of FUT8 differentiating LUAD from normal lung tissues (Figure 3B). The AUC was .919 (95% CI = .897–.941, *p* < .001) with a specificity of 98.3% at a sensitivity of 79.2%.

### Survival Analysis of Fucosyltransferase 8 in Lung Adenocarcinoma Using Public Databases

The Kaplan-Meier plotter was used to evaluate the association between FUT8 expression and survival using GEO and TCGA datasets. A total of 672 LUAD patients with FUT8 expression and survival data were categorized into high expression group and low expression group according to the median of all samples. We found that FUT8 overexpression was correlated with worse OS in LUAD patients (*p* = .00015; HR = 1.62; 95% CI = 1.26–2.08) (Figure 3C). The median survival of LUAD patients with low FUT8 expression was 136.33 months, while high FUT8 expression cohort had a median survival of 75.43 months.

### Expression Validation of Fucosyltransferase 8 in Lung Adenocarcinoma Tissues by IHC

**FIGURE 4:**
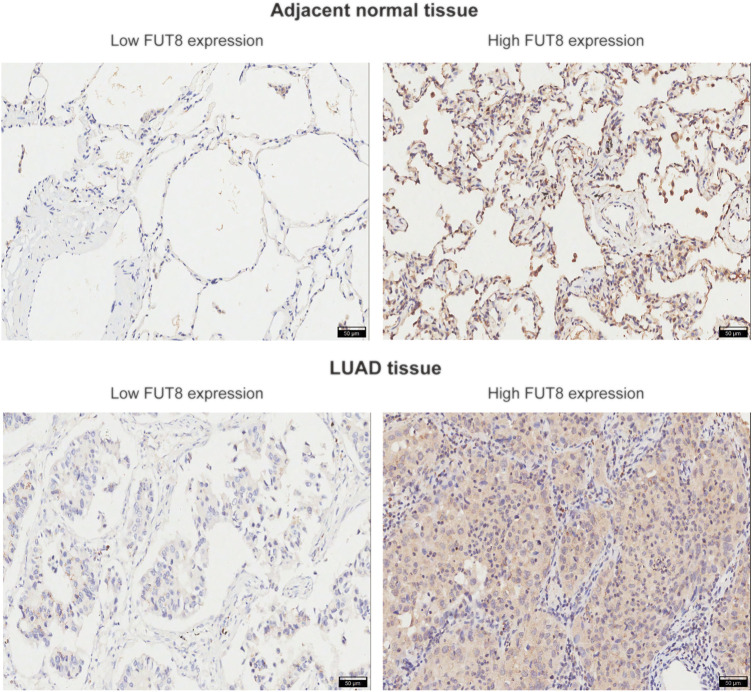
Representative images of FUT8 IHC staining in adjacent normal and LUAD tissues are shown. Scale bar = 50 μm. Abbreviations: FUT8, fucosyltransferase 8; LUAD, lung adenocarcinoma.

To verify the results from public databases, we examined FUT8 expression in LUAD tissues and their matched adjacent normal tissues by IHC. As shown in Table 2, the overexpression rate of FUT8 protein was significantly higher in LUAD (52/92, 56.5%) compared to normal tissues (14/88, 15.9%) (*p* = 1.585 × 10^−8^). The representative pictures of FUT8 immunohistochemical expression in LUAD and normal samples are shown in Figure 4. The correlations between FUT8 protein expression and clinicopathological features was further investigated in 92 patients with LUAD. The result showed that FUT8 expression was not associated with age, sex, differentiation, tumor status, clinical stage, lymph node metastases and EGFR mutation (all *p* > .05; Table 3).

**TABLE 2:** Analysis of FUT8 protein expression in LUAD and adjacent normal tissues.

	Case	FUT8 protein expression		*p*-value
		Low (%)	High (%)	
LUAD tissues	92	40 (43.5%)	52 (56.5%)	1.585 × 10^−8^
Adjacent normal tissues	88	74 (84.1%)	14 (15.9%)	

FUT8, fucosyltransferase 8; LUAD, lung adenocarcinoma.

**TABLE 3:** Correlation between FUT8 expression and clinicopathological factors in 92 patients with LUAD.

Characteristics	FUT8 protein expression		*p*-value
	Low (*N* = 40)	High (*N* = 52)	
Age, years	61.35 ± 9.01	64.23 ± 10.44	.168
Sex			
Male	23 (42.5%)	28 (46.2%)	.727
Female	17 (57.5%)	24 (53.8%)	
Differentiation			
Well/moderate	28 (70.0%)	29 (55.8%)	.163
Poor	12 (30.0%)	23 (44.2%)	
Tumor status (T)			
T1+T2	29 (72.5%)	44 (84.6%)	.155
T3+T4	11 (27.5%)	8 (15.4%)	
Stage			
I + II	27 (67.5%)	32 (61.5%)	.555
III + IV	13 (32.5%)	20 (38.5%)	
Lymph node metastasis			
Negative	22 (55.0%)	21 (40.4%)	.164
Positive	18 (45.0%)	31 (59.6%)	
EGFR mutation			
Negative	33 (82.5%)	38 (73.1%)	.286
Positive	7 (17.5%)	14 (26.9%)	

Values are mean ± SD (standard deviation) or n (%).

FUT8, fucosyltransferase 8; LUAD, lung adenocarcinoma; EGFR, epidermal growth factor receptor.

### Association Between Fucosyltransferase 8 Expression and Survival in Lung Adenocarcinoma Patients

**FIGURE 5:**
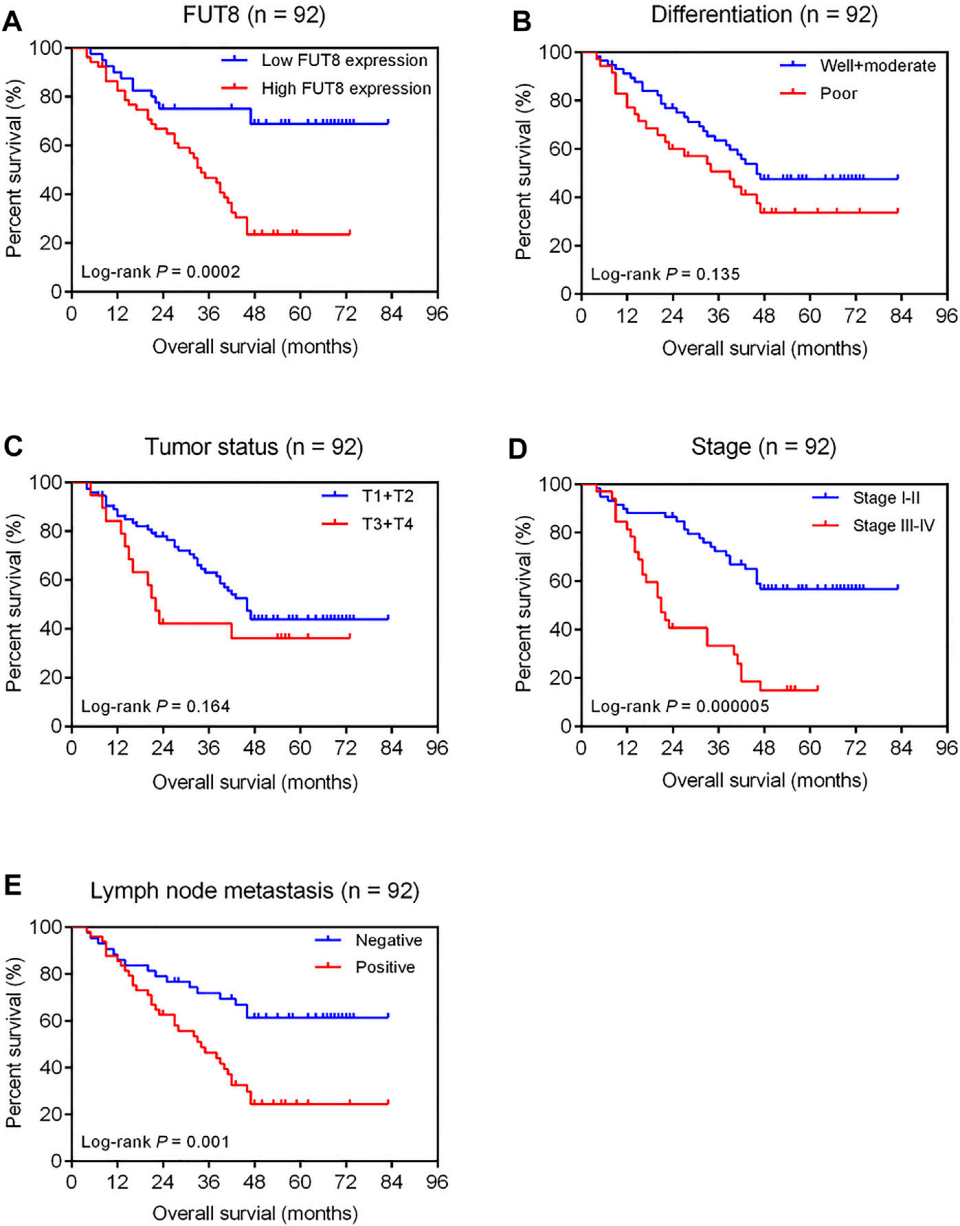
Kaplan-Meier analysis of overall survival of 92 LUAD patients associated with FUT8 expression **(A)**, differentiation **(B)**, tumor status **(C)**, clinical stage **(D)** and lymph node metastasis **(E)**, respectively. Abbreviations: LUAD, lung adenocarcinoma; FUT8, fucosyltransferase 8.

Figure 5A shows the Kaplan-Meier survival curve for OS for high and low FUT8 expression groups. The LUAD patients with low FUT8 expression showed significantly better OS than those with high FUT8 expression (*p* = .0002). We also assessed the prognostic significance of clinicopathological features for survival of patients with LUAD (Figure 5). The results revealed that clinical stage and lymph node metastasis correlated to OS of LUAD patients. The patients with advanced stage LUAD demonstrated significantly worse prognosis than those with early stage LUAD (*p* = .000005; Figure 5D). The lymph node-positive patients showed significantly worse OS than those with negative lymph node metastasis (*p* = .001; Figure 5E).

Univariate analysis revealed that high FUT8 expression (HR = 3.27; 95% CI = 1.70–6.30; *p* = .0004), advanced stage (HR = 3.45; 95% CI = 1.95–6.08; *p* = .00002), and positive lymph node metastasis (HR = 2.55; 95% CI = 1.40–4.65; *p* = .002) were associated with shorter OS (Table 4). After accounting for the effects of age, sex, differentiation, tumor status, stage and lymph node metastasis, multivariate Cox regression analysis indicated that the level of FUT8 expression was an independent predictor of poor OS (HR = 2.87; 95% CI = 1.42–5.77; *p* = .003; Table 4). Additionally, clinical stage was also an independent prognostic factor (HR = 2.81; 95% CI = 1.36–5.83; *p* = .005; Table 4).

**TABLE 4:** Univariate and multivariate Cox regression analyses for overall survival in 92 LUAD patients.

Characteristics	N (%)	Univariate analysis		Multivariate analysis	
		HR (95% CI)	*p*-value	HR (95% CI)	*p* Value
FUT8 expression, high vs. low	52/40 (56.5%/43.5%)	3.27 (1.70–6.30)	.0004*	2.87 (1.42–5.77)	.003*
Age, > 60 vs. ≤ 60 years	54/38 (58.7%/41.3%)	1.29 (.73–2.28)	.384	1.24 (.68–2.27)	.478
Sex, male vs. female	51/41 (55.4%/44.6%)	1.44 (.82–2.54)	.205	1.51 (.84–2.73)	.173
Differentiation, poor vs. well/moderate	35/57 (38.0%/62.0%)	1.52 (.87–2.66)	.140	1.09 (.59–2.00)	.787
Tumor status, T3+T4 vs. T1+T2	19/73 (20.7%/79.3%)	1.58 (.82–3.02)	.171	.93 (.42–2.08)	.861
Stage, III + IV vs. I + II	33/59 (35.9%/64.1%)	3.45 (1.95–6.08)	.00002*	2.81 (1.36–5.83)	.005*
Lymph node metastasis, positive vs. negative	49/43 (53.3%/46.7%)	2.55 (1.40–4.65)	.002*	1.58 (.77–2.36)	.213

*p < .05.

LUAD, lung adenocarcinoma; FUT8, fucosyltransferase 8; HR, hazard ratio; CI, confidence interval.

### Knockdown of Fucosyltransferase 8 Inhibits Lung Adenocarcinoma Cell Proliferation and Triggers Cell Apoptosis

**FIGURE 6:**
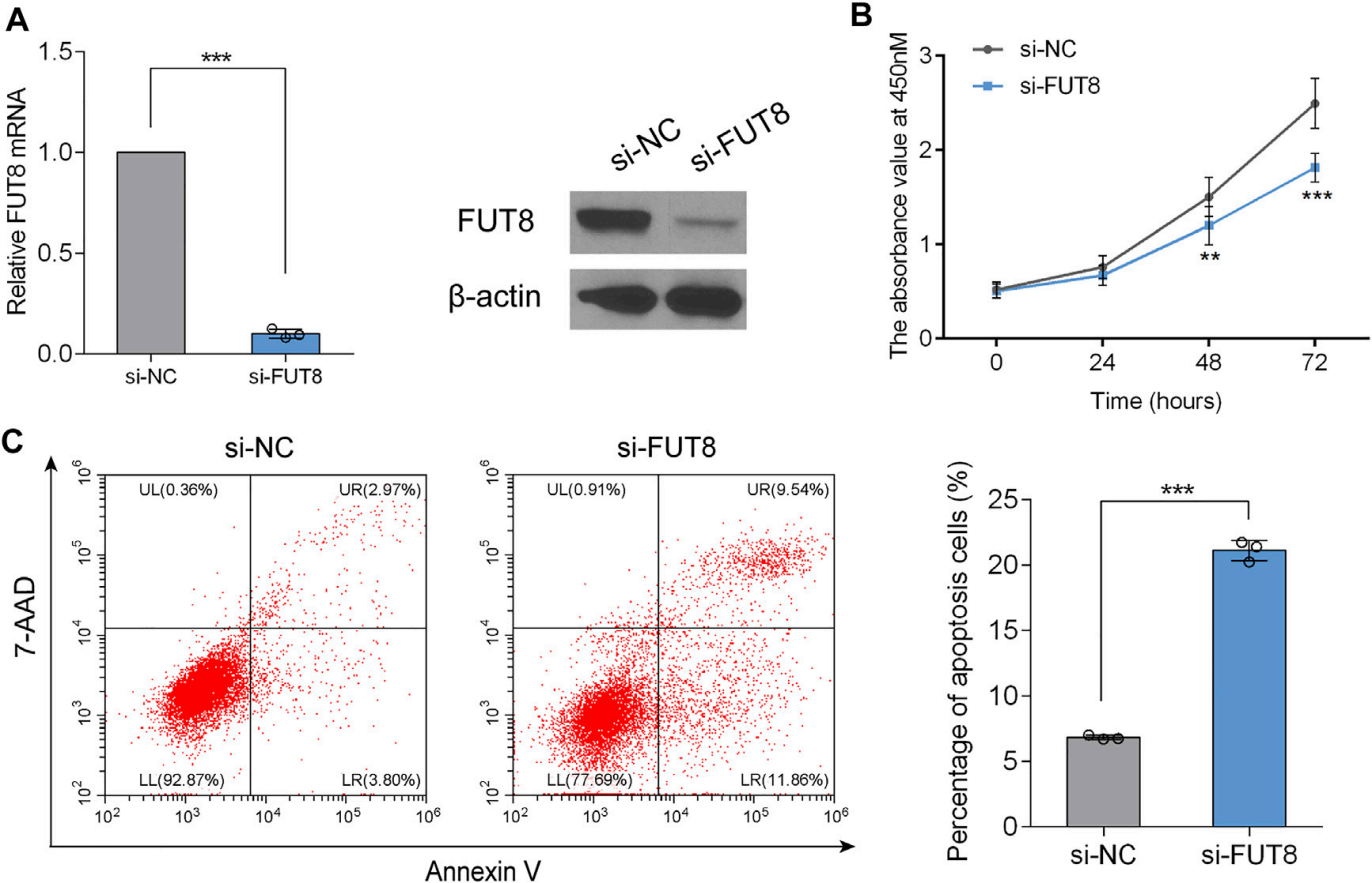
FUT8 knockdown restrained proliferation and induced apoptosis in A549 cells. **(A)** The expression levels of FUT8 in A549 cells transfected with si-FUT8 or si-NC detected by RT-PCR (left panel) and western blot (right panel). **(B)** The effect of FUT8 knockdown on the cell growth in A549 cells measured by CCK-8 assay. Each point represents the mean from duplicate wells in four independent experiments. **(C)** Annexin V and 7-AAD staining showing the percentage of apoptosis cells in si-FUT8 and si-NC transfected A549 cells. Each column represents the mean from three independent experiments. All values shown were mean ± SD. **p < .01, ***p < .001. Abbreviations: FUT8, fucosyltransferase 8; NC, negative control.

To determine the biological role of FUT8 in LUAD cell proliferation and apoptosis, we established FUT8-knockdown cell lines (A549) using siRNA. RT-PCR and western blot analysis indicated that the expression of FUT8 was dramatically down-regulated on the mRNA and protein level by FUT8 siRNA (Figure 6A). As shown in Figure 6B, silencing the expression of FUT8 *in vitro* markedly arrested cell proliferation in LUAD cells. In addition, LUAD cells with down-regulated expression of FUT8 showed a significantly increased percentage of apoptotic cells (*p* < .001; Figure 6C).

## Discussion

The up-regulation of FUT8 expression has been reported in various types of cancer, including pancreatic, colorectal, thyroid, ovarian and lung cancers, and linked to the severity of malignant tumors (22–25). For example, in pancreatic ductal adenocarcinoma, FUT8 protein expression was elevated in carcinoma compared with normal pancreatic duct tissues, and FUT8 overexpression was directly correlated with lymph-node metastases and worse relapse-free survival (22). In colorectal carcinoma, a significant increase was observed in the FUT8 expression in tumor tissues as compared to healthy and transitional tissues, inflammatory lesions and adenomas (24). A study by Chen et al. investigated the clinical relevance of FUT8 expression in NSCLC, and indicated up-regulation of FUT8 in NSCLC was associated with distal metastasis, recurrence, and poor survival of patients (26). Another study on NSCLC by Honma et al. showed that high expression of FUT8 was found in 67 of 129 NSCLC patients, and FUT8 expression was significantly more prevalent in non-squamous cell carcinoma than in squamous cell carcinoma cases (21). Zhou et al. (27) conducted RT-PCR and western blot analyses in 13 and 9 LUAD tissues, respectively, and found that the mRNA and protein expression level of FUT8 was significantly increased in LUAD. In the present study, we systemically retrieved previous microarray data in GEO for a meta-analysis to retrospectively investigate the expression levels of FUT8 in LUAD. The pooled results showed that FUT8 was significantly up-regulated in LUAD tissues, and FUT8 expression had a potential diagnostic value for LUAD. The results above were further validated by TCGA data mining. According to the survival analyses using Kaplan-Meier plotter, in LUAD patients, the death rate tended to be higher and the prognosis was likely to be worse under high FUT8 expression.

In this study, the FUT8 expression and its value as a prognostic factor was further confirmed using LUAD specimens and adjacent lung tissues recruited from our hospital. The results of IHC analysis showed FUT8 was indeed overexpressed in LUAD tissues. In a previous study, we investigated the profile of serum *N*- and *O*-glycans in NSCLC patients and control individuals using lectin microarrays (18). We observed that the serum levels of α1,6-fucosylation identified by *Aleuria aurantia* Lectin (AAL, a lectin prefers to bind core fucosylation) were elevated in LUAD patients. These results suggested that the increased levels of core fucosylation observed in serum of patients with LUAD might be the result of increased synthesis of core fucosylated proteins in the cancer tissue. In addition, Kaplan-Meier survival curves suggested that increased FUT8 expression was associated with poor prognosis in patients with LUAD. More importantly, FUT8 expression was significantly correlated with OS of LUAD patients both in univariate analysis and multivariate analysis. The result indicated FUT8 expression was an independent risk indicator of OS in LUAD. We also carried out *in vitro* experiments to uncover the biological function of FUT8 in LUAD. We found that knockdown of FUT8 in LUAD cells markedly repressed cell proliferation and induced cell apoptosis, indicating that FUT8 could drive LUAD development.

There are still some limitations in this study. Due to the relatively small sample size for IHC analysis, further studies with larger cohort of patients are required to validate our findings. Further experiments are indispensable to understand the underlying mechanism of FUT8 promoting LUAD development. Furthermore, core fucosylation of glycoproteins, such as adhesions, integrins and TGF-β1 (11, 28–30), might be associated with the migratory and invasive ability of LUAD. Further investigations focused on the functions of FUT8 in LUAD migration and invasion are needed to conclusively determine its biological role.

In summary, FUT8 is up-regulated in LUAD tissues and increased FUT8 expression is correlated with poor prognosis of LUAD patients. *In vitro* studies show that FUT8 might facilitate cell proliferation and inhibit apoptosis in LUAD. These findings indicate that FUT8 might be used as a novel independent marker reliably predicting the clinical outcome in LUAD patients, and has the potential to become a valuable therapeutic target for LUAD.

## Data Availability

The original contributions presented in the study are included in the article/Supplementary Material, further inquiries can be directed to the corresponding author.
