# Supplementary material for: Fucosyltransferase 8 is Overexpressed and Influences Clinical Outcomes in Lung Adenocarcinoma Patients
Source: Pathol Oncol Res. 2022 Feb 14;28:1610116. doi: 10.3389/pore.2022.1610116 (PMC8883820; doi:10.3389/pore.2022.1610116)
Supplement: Supplementary file 1 [file Table1.DOCX]

Table S1. The primers used in RT-PCR analysis for FUT8 and β-actin.

| Gene | Forward（5’-3’） | Reverse（5’-3’） |
| --- | --- | --- |
| FUT8 | GTGGTGGGTGTCTCAGTTTGT | CTGGATGTTTGAAGCCAAGC |
| β-actin | ACAACCGCATGTCCAGAAACG | GTAACGGCTGTCCTACGT |

Abbreviations: FUT8, fucosyltransferase 8.
